# Liquid marble technology to create cost-effective 3D cardiospheres as a platform for *in vitro* drug testing and disease modelling

**DOI:** 10.1016/j.mex.2020.101065

**Published:** 2020-09-12

**Authors:** Jeffrey Aalders, Laurens Léger, Tim Tuerlings, Sergio Ledda, Jolanda van Hengel

**Affiliations:** a Medical Cell Biology research group, Department of Human Structure and Repair, Faculty of Medicine and Health Sciences, Ghent University, Corneel Heymanslaan 10, Building B, Entrance 36, 9000 Ghent, Belgium; b Department of Veterinary Medicine, University of Sassari, Sassari, Italy

**Keywords:** Cardiomyocytes, Human stem cells, Cell culture techniques, *In vitro* modelling, Liquid marbles

## Abstract

Three-dimensional (3D) cell culturing has several advantages over 2D cultures. 3D cell cultures more accurately mimic the *in vivo* environment, which is vital to obtain reliable results in disease modelling and toxicity testing. With the introduction of the Yamanaka factors, reprogramming of somatic cells to induced pluripotent stem cells (iPSCs) became available. This iPSC technology provides a scalable source of differentiated cells. iPSCs can be programmed to differentiate into any cell type of the body, including cardiomyocytes. These heart-specific muscle cells, can then serve as a model for therapeutic drug screening or assay development. Current methods to achieve multicellular spheroids by 3D cell cultures, such as hanging drop and spinner flasks are expensive, time-consuming and require specialized materials and training. Hydrophobic powders can be used to create a micro environment for cell cultures, which are termed liquid marbles (LM). In this procedure we describe the first use of the LM technology for 3D culturing *in vitro* derived human cardiomyocytes which results in the formation of cardiospheres within 24h. The cardiospheres could be used for several in depth and high-throughput analyses.

## Graphical abstract

**Figure:**
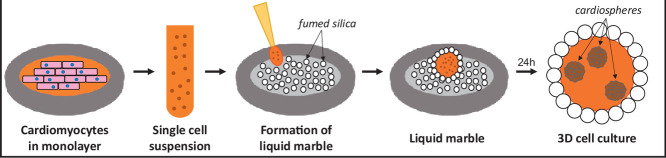

Specifications table

**Table:** 

Subject Area	Pharmacology, Toxicology and Pharmaceutical Science
More specific subject area	*In vitro* cardiac modeling
Method name	Liquid marble technology
Name and reference of original method	*-*
Resource availability	*-*

## Introduction

With the introduction of the Yamanaka factors to generate human induced pluripotent stem cells (hiPSCs) from somatic cells, an attractive alternative for animal models was established [1]. hiPSCs are particularly interesting because they can divide indefinitely and provide a practically unlimited source for all cells of the human body. Moreover, a single urine sample is sufficient as starting material for reprogramming to hiPSCs, thus minimizing invasive procedures for patients, but still providing a patient-specific cell source [2]. The hiPSC technology is a useful tool in toxicity testing and disease modelling, allowing high-throughput analysis which opens up possibilities for personalized medicine approaches.

Laboratory animals offer a number of advantages and are extensively used to model human diseases and drug responses. Selective breeding and genetic manipulation of mice have made many different genotypes and phenotypes available for research. However, in many cases, mouse and other animal models have failed to be predictive in pre-clinical drug toxicity testing. Early and reliable safety screening of novel drug candidates in the discovery process is a prerequisite to improve drug performance and reduce costs. Unfortunately, previously undetected side effects of several drug compounds, leading to increased risk for morbidity and mortality have resulted in discontinuation and withdrawal from the market, imposing financial drawbacks to pharmaceutical companies.

Functional contracting cardiomyocytes (CM) can be efficiently derived from hiPSCs and are being used in human *in vitro* cardiac models for pre-clinical drug assessments. Several high-throughput screening techniques have been established for *in vitro* CMs, including multi electrode array (MEA), video based motion analysis, cellular impedance and calcium transient imaging. MEA measures the extracellular field potential (EFP) which corresponds to an *in vitro* ECG and allows detection of arrhythmogenic drug effects as demonstrated in CiPA, a blinded multi-center study [3]. Video analysis of contracting CMs using the Musclemotion software allows tracking of the physical movement of CMs [4]. A combinatory approach using these techniques, fully covering effects on the excitation-contraction characteristics of CMs, was determined as an ideal platform for drug toxicity testing in the CSAHi HEART Initiative [5].

While the majority of cell models are described as two-dimensional (2D) monolayer cultures, the field of *in vitro* cardiac modelling is rapidly advancing and shifting to three-dimensional (3D) cell culture systems. 3D models closely mimic the *in vivo* environment, modeling the complex cell interactions more accurately, especially in terms of structural and functional properties. Moreover, the 3D cell-based assay could contribute to the reduction of experimental animal studies, and hence the cost of subsequent screening processes. Various 3D culture techniques are at hand, both scaffold based and scaffold-free [6]. Scaffold-free techniques include hanging drop, spinner flask, centrifuge-forced aggregation and suspension cultures on low-adherence surfaces. However, the majority of the 3D technologies require specialized and expensive equipment, extensive experience, are time-consuming and have a relatively low reproducibility.

In this protocol we describe a robust and cost-effective method for the generation of 3D *in vitro* cardiac cultures that does not require specialized equipment. Liquid marbles (LM) can be produced by coating a small liquid droplet with a hydrophobic powder, providing a confined microenvironment with a non-adhesive, transparent and gas permeable shell. Silica nanoparticles can be used to make these LM, being hydrophobic, biocompatible and chemically very stable. The use of hydrophobic powders in LM was first described by Aussillous and Quéré in 2001 [7]. This technology provides a method to create a micro bioreactor for 3D cell culture. Cells in this non adherent liquid surface tend to rapid self-assemble into spheroid structures at the concave bottom of the marble as a consequence of integrated spinning and stationary cues. The transparent nature of the LM allows for easy visual assessment of the cells. Other advantages include low cost, self-repairing ability of the LM coating, low cell culture medium consumption, high reproducibility and gas permeability [8]. More advanced approaches with the LM technology have been described. These include the floating LM [9] and a hydrogel sphere consisting of agarose with growth factors [10], both reducing evaporation of the LM and improving the reproducibility of the spheroid shape.

The scaffold-free approach for 3D cell culture compartmentalization using LM can serve as a suitable screening platform for compounds and requires only a small volume of liquid. The generated 3D cardiospheres described in this protocol would provide interesting material for semi-automated high-throughput drug screening and offers a cost-effective alternative. For instance, the sphericity, cell area and cell viability can be determined using microscopy images and can be a possible read-out to assess toxic effects. Moreover, LM are also of interest in disease modelling containing more cell types. Another alternative use of LM could be in the *in vitro* cardiac differentiation of hiPSCs, previously it was reported the LM was suitable for spontaneous cardiac differentiation in murine embryonic stem cells [11]. Although not assessed in this protocol, it may enhance differentiation protocols [12]. Applications of the LM have extended to the broader field of biology including the use in enhancing polymerase chain reactions [13].

## Methods

### Ethics statement

Experiments with hESCs and hiPSCs were approved by the local ethical committee of Ghent University Hospital (EC UZG 2017/0855).

### Cell culture

The human embryonic stem cell (hESC) line H9 (WA09, WiCell) was cultured feeder-free in Essential 8™ medium (Life Technologies, cat no. A1517001) supplemented with Penicillin/Streptomycin (100 u/ml Penicillin and 100 µg/ml Streptomycin, Life Technologies, cat no. 15140-122) on Geltrex™ coating (Life Technologies, cat no. A1413302) in 6-well and 12-well CELLSTAR® culture plates (Greiner Bio One, cat no. 657160 and cat no. 665180). All cell cultures were maintained at 37 °C, in a humidified atmosphere of 5% CO_2_ and 5% O_2_. Phase-contrast images of cell cultures were made using EVOS™ XL Core Cell Imaging System (Thermo Fisher Scientific).

### Differentiation to cardiomyocytes

To derive CMs from hESCs, a protocol previously reported by Lian *et al* based on modulation of Wnt was used [14]. In short, 80% confluent hESCs cultures were dissociated into a single cell suspension using TrypLE (Life Technologies, cat no. 12604013) for 5 min at 37 °C. TrypLE was inactivated with Essential 8 medium and the cell suspension was centrifuged for 5 min at 200 g. The pellet was resuspended in E8 medium supplemented with 1:100 RevitaCell (Life Technologies, cat no. A2644501) and transferred to a Geltrex-coated 12-well (200.000 hESCs/ well). The following days, the medium was refreshed daily with Essential 8 medium until a confluency of 80% (approx. 3 days) was reached. At day 0, the cells were washed with PBS and the medium was changed to cardio differentiation medium supplemented with 4 µM CHIR99021 (Merck, cat no. 361559). Cardio differentiation medium consists of RPMI 1640 with HEPES (5958 mg/l) and GlutaMAX (L-Alanyl-Glutamine 446 mg/l) (Life Technologies, cat no. 72400-021) supplemented with 0.125 mg/ml Albumin (Sigma-Aldrich, cat no. A9731) and 0.05 mg/ml L-Ascorbic Acid 2-Phosphate (Sigma-Aldrich, cat no. A8960). After exactly 48h (day 2), the cells were washed with PBS and medium was changed to cardio differentiation medium supplemented with 5 µM IWP2 (Merck, cat no. 681671). The medium was changed to cardio differentiation medium on day 4 and day 6. On day 8 and every other day, the medium was refreshed with cardio maintenance medium. Cardio maintenance medium consists of RPMI 1640 with HEPES and GlutaMAX supplemented with 1:100 B27 supplement with insulin (Life Technologies, cat no. 17504-044).

### Making a single cell suspension of cardiomyocytes

To dissociate the monolayer CMs (Fig. 2B) into a single cell suspension, the Multi Tissue Dissociation Kit 3 (Miltenyi Biotec, cat no. 130-110-204) was used. In short, the cell culture was washed three times with PBS. Then cell cultures were incubated for 10 min at 37 °C with 400 µl dissociation mix (consisting of 40 µl enzyme T and 360 µl buffer X) per 12-well. Cells were gently pipetted up and down (approx. 3–5 times) to dissociate using a 1 ml micropipette. 600 µl cardio maintenance medium supplemented with 20% FBS was added per 12-well. The cell suspension was collected and transferred to a 70 µm strainer. The 12-well was washed with another 2 ml of cardio maintenance medium supplemented with 20% FBS and transferred to the same strainer. At this point a small sample could be taken to determine the number of cells using Trypan blue.

### Seeding of cardiomyocytes

The single cell suspension was centrifuged for 5 min at 200 g. The pellet was resuspended in cardio maintenance medium supplemented with 20% FBS and 1% RevitaCell to have a final solution between 500–2000 CMs/µl. The cell suspension was transferred to a 50 µl droplet of 1:10 Geltrex in a 12-well that was incubated for 1h at 37 °C, and half of the droplet (25 µl) was removed just before applying 25-100 µl cell suspension the small coated area (this allows high-density seeding of CMs and increases cell viability). After 1h of additional incubation at 37 °C (allowing CM attachment), 1 ml cardio maintenance medium supplemented with 20% FBS and 1% RevitaCell was carefully added. After two days, the medium was replaced by normal cardio maintenance medium and refreshed every other day.

### Purification of cardiomyocytes (optional)

If the percentage of cardiomyocytes in the population is insufficient, purification using the human PSC-Derived Cardiomyocyte Isolation Kit (Miltenyi Biotec, cat no. 130-110-188) can be performed. The isolation can be performed as a single step procedure with negative selection for CMs for up to 5 × 10^6^ cells. To increase cell viability, it is recommended to perform the majority of procedures on ice. In short, a single cell suspension was made as described in 2.4 and centrifuged for 5 min at 200 g. The pellet was dissolved in 80 µl wash buffer (consisting of PBS supplemented with 2% FBS) and 20 µl Non-Cardiomyocyte Depletion Cocktail was added, mixed and incubated at 4 °C for 5 min. Next, the cells were washed with 1 ml wash buffer and centrifuged for 5 min at 200 g. The pellet was dissolved in 80 µl wash buffer. 20 µl Anti-Biotin MicroBeads were added, mixed and incubated for 10 min at 4 °C. 400 µl wash buffer was added to the cell suspension. An LS column was placed in the magnetic field of a MACS separator. The column was washed with 3 ml wash buffer. The cell suspension (500 µl) was added to the column and the flow-through was collected. The column was washed three times with 3 ml wash buffer, flow-through was collected in the same collection tube. The flow-through contains the purified CM cell suspension.

### Production of liquid marbles

**Fig. 1:**
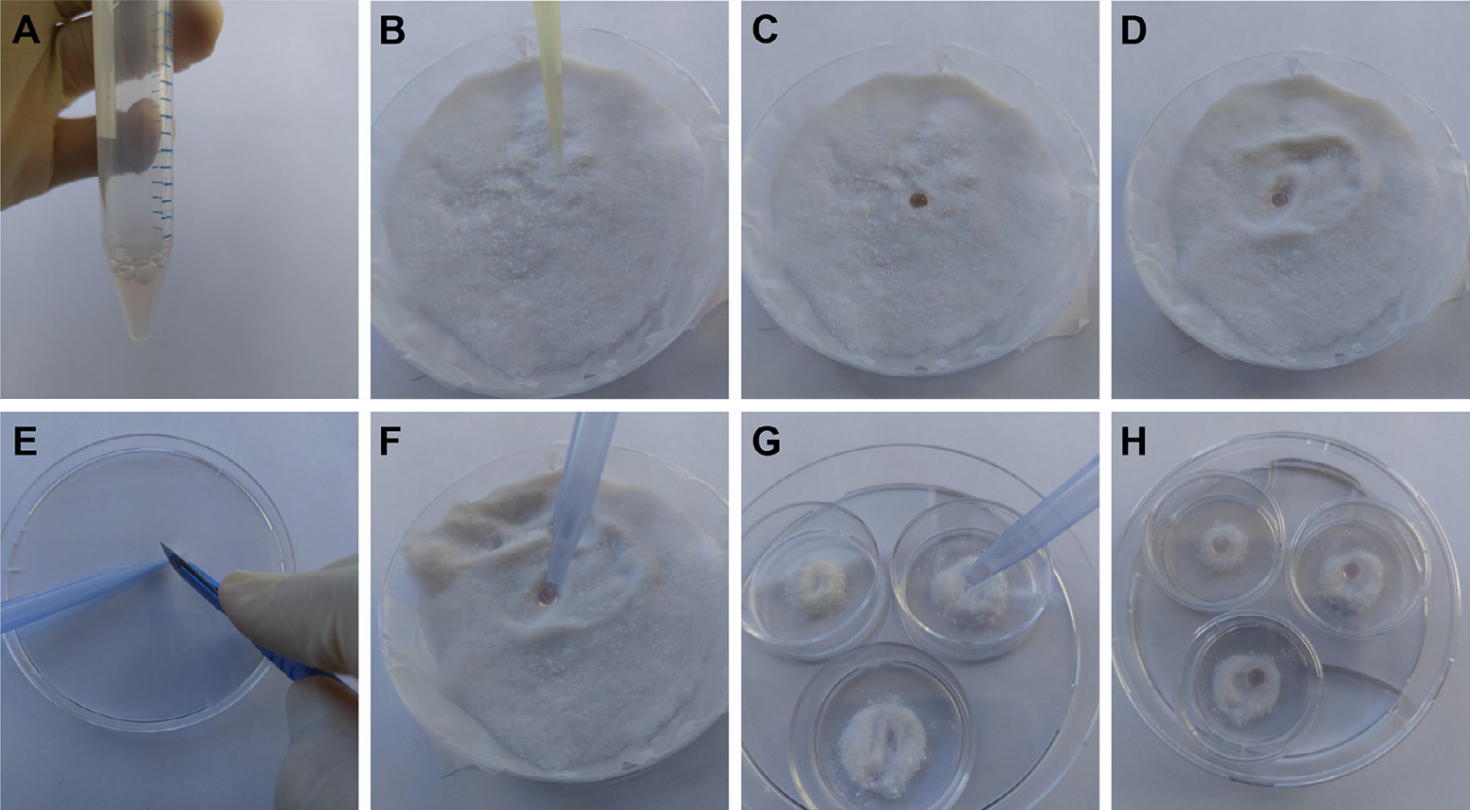
Production of the liquid marble bioreactor. (A) Single cell suspension is prepared. (B) Cell suspension is transferred to hydrophobic silica powder on a parafilm-covered petri dish. (C) Cell suspension is placed on powder bed and forms a droplet. (D) Rolling around of Liquid Marble (LM) to ensure even coating. (E) Adapting 1000 µl pipette tip with a scalpel. (F) Pickup liquid marble using modified 1000 µl pipette tip. (G) Transfer to 25 mm petri dish containing silica powder. (H) Placement into larger petri dish filled with PBS. (For interpretation of the references to color in the text, the reader is referred to the web version of this article.)

**Fig. 2:**
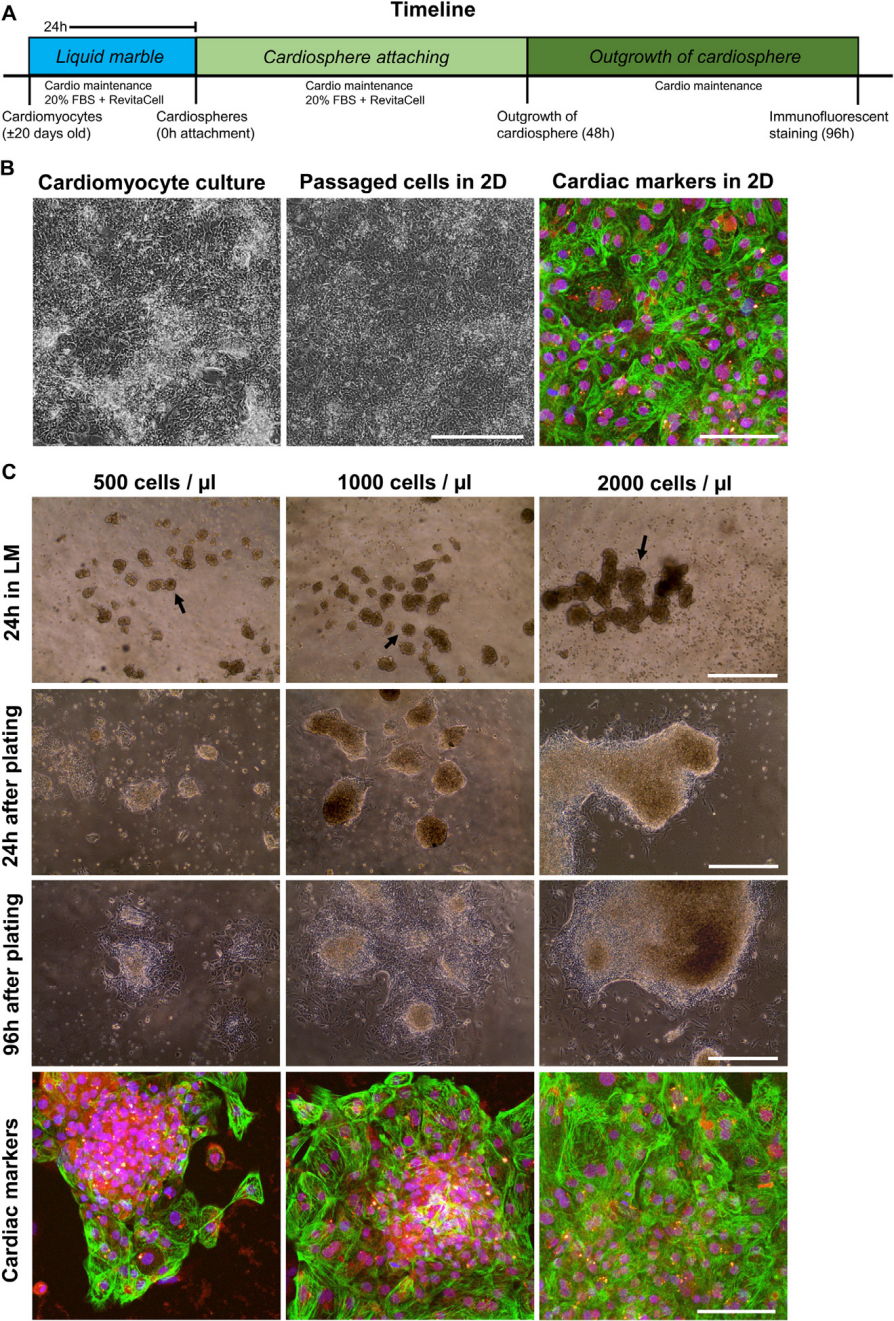
*In vitro* 2D and 3D cardiomyocyte cultures. (A) Timeline of the presented liquid marble procedure from start (0h) making the marble, until 96h after attachment of cardiosphere used for immunofluorescent staining. (B) 20-day-old cardiomyocytes grown in 2D were passaged to a glass coverslip and immunofluorescent staining after 96h of attachment shows expression of cardiac markers cTnT (green), NKX2.5 (red), a counterstain with Hoechst (blue) was performed. (C) Cardiospheres (indicated by the black arrow) were produced after 24h in a 50 µl Liquid Marble (LM) of different cardiac culture concentration (500, 1000 and 2000 cells/ µl). Phase-contrast pictures show attachment after cardiosphere harvesting after 24h and 96h. Maximal intensity projection images show cardiac marker expression for cTnT (green) and NKX2.5 (red) and nuclei (blue). Bar in phase-contrast images indicates 500 µm, and 100 µm in confocal images. (For interpretation of the references to color in the text, the reader is referred to the web version of this article.)

All steps (Fig. 1) were performed in a biosafety cabinet to maintain sterile conditions and to avoid inhalation of the fine powder. Fig. 2A shows the timeline for the experiment, which starts at 0h with the production of LM.

1. The surface of a 9 cm petri dish was covered with parafilm M®.
2. A layer (of ± 1 cm) fumed silica powder (Cabot Corp, Cab-O-Sil, TS-530) was evenly distributed over de surface of the parafilm.
3. A droplet (25-100 µl) of cell suspension (500, 1000 and 2000 cells/ µl) was carefully placed on top of the powder bed.
4. The droplet was rolled around in circular motions in the dish, ensuring that the droplet is evenly coated with the powder, creating a LM.
5. Before transferring the LM, a small amount of silica powder was placed in the recipient non-adherent 25 mm petri dish (Greiner Bio One, 627102).
6. Transfer of the LM was done using a 1000 µl micropipette, of which the tip was cut so that its diameter is slightly smaller than the diameter of the marble, ensuring a proper grip (tip: use a scalpel instead of scissors, this prevents sharp edges on the pipette tip).
7. In addition, the inside of the modified pipette tip was coated with a small amount of silica powder to avoid adhesion of the LM to the pipette tip during transferring.
8. The LM was then sucked up into the modified tip and gently placed on the small amount of silica powder in the recipient 25 mm petri dish.
9. The 25 mm dishes (3x) containing one LM each were placed into a 9 cm petri dish. To avoid evaporation, the 9 cm petri dish was filled with PBS (± 7 ml).
10. The LM was incubated for 24h at 37 °C.

### Cardiosphere harvesting

1. Following incubation of the LM for 24h, the spheres were harvested.
2. To prepare the recipient, a coating of 50 µl 1:10 Geltrex was incubated for 1h at 37 °C. It is recommended to create a humid environment by adding sterilized water in the surrounding well spaces, this helps minimizing evaporation. For immunofluorescent staining, this step can be executed on glass coverslips.
3. Just before plating the LM (with 25–100 µl volume), half of the Geltrex coating droplet (25 µl) was removed.
4. The LM was transferred using a 1000 µl micropipette, of which the tip was cut with a scalpel so that its diameter is slightly smaller than the diameter of the marble.
5. The LM was then sucked up into the modified tip and gently transferred to the recipient containing the Geltrex droplet. By slowly placing the LM on top of the Geltrex droplet, the marble breaks, causing dispersion of the contents in the droplet.
6. The cardiospheres in the new recipient were incubated for 1h at 37 °C.
7. Additional cardio maintenance medium supplemented with 20% FBS and 1% RevitaCell was carefully added to the well (1 ml/ 12-well).
8. The attached cardiospheres were incubated for 48h at 37 °C to further attach and spread out.
9. After 48h, the medium is changed every other day with cardio maintenance medium.

### Immunohistochemical analysis of cardiac cultures

Cell cultures were fixed using 4% PFA for 20 min. Cells were permeabilized for 30 min with 0.1% Triton X-100 diluted in PBS. The fixed cells were incubated with blocking solution consisting of 5% Goat serum (Life Technologies, cat no. 16210-064) in PBS for 30 min. Next, the cells were incubated overnight at 4 °C with primary antibodies (Table 1) diluted in PBS containing 0.05% Tween20 and 1% bovine serum albumin (BSA). The next day, the cells were incubated for 60 min at RT with secondary antibodies (Table 1) diluted in PBS containing 0.05% Tween20 and 1% BSA and subsequently incubated for 10 min with 0.1% Hoechst solution. The stained cells were mounted and images were made using the ZEISS LSM900 confocal microscope.

**Table 1:** Antibodies used for immunofluorescent staining.

Staining products	Dilution	Company (cat no.)	Remarks
Rabbit-anti-NKX2.5	1/100	Life Technologies (701622)	Primary Ab
Mouse-anti-TNNT2	1/250	Life Technologies (MA5-12960)	Primary Ab
Goat-anti-Mouse IgG Dylight 488	1/500	Life Technologies (35503)	Secondary Ab
Goat-anti-Rabbit IgG Dylight 594	1/500	Life Technologies (35561)	Secondary Ab

## Results

Different volumes of cardiomyocyte cell suspensions were used (25, 50 and 100 µl) to make LM in this protocol. All these volumes are suitable for the production of cardiospheres. At the small volume (25 µl), more care for evaporation has to be taken. The cell suspension concentration had an influence on the formation of cardiospheres. As illustrated in Fig. 2C with marbles of 50 µl size, all concentrations led to the formation of 3D aggregates. However, in the 2000 cells/ µl concentration, the cardiospheres tended to aggregate with each other. The 500 cells/ µl concentration resulted in relatively smaller cardiospheres compared to the 1000 and 2000 cells/ µl conditions. After harvesting, the cardiospheres were seeded on a glass coverslip. Immunofluorescent staining revealed that cardiac markers were retained after 3D culture in LM for all conditions (Fig. 2C) similar as cardiomyocytes cultured in 2D (Fig. 2B). Depending on the experimental needs and the properties of the cell line, optimal cell suspension concentrations may vary. In this protocol we describe the LM technique with the broadly used H9 line that in our hands works comparable for human induced pluripotent stem cells (hiPSCs).

**Fig. 3:**
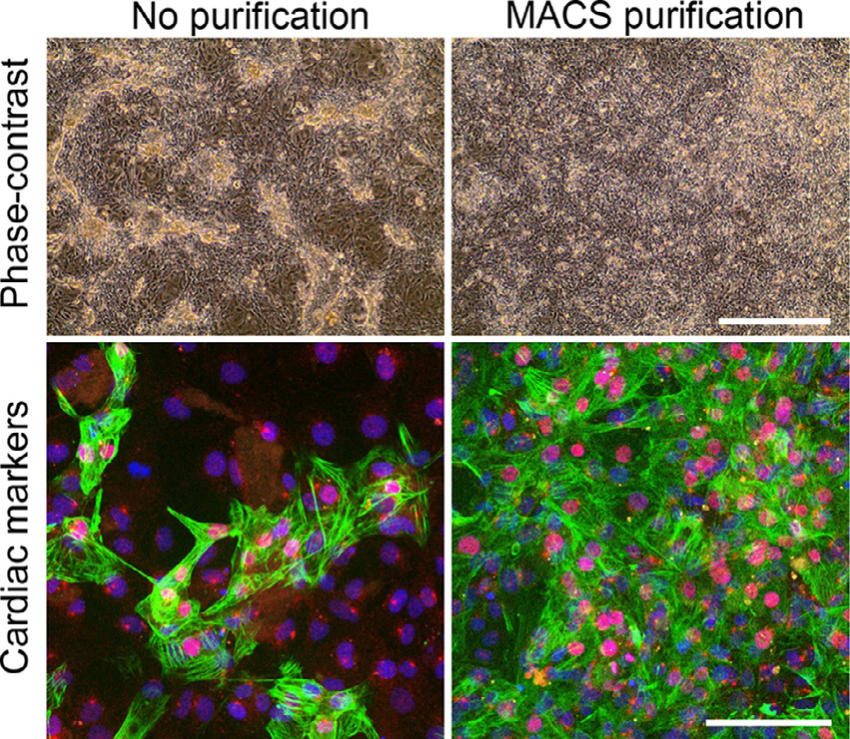
Purification of cardiac cultures using MACS. Phase contrast images show 2D cardiac cultures 48 h after passaging without purification and with MACS purification. Cardiac markers cTnT (green) and NKX2.5 (red) are showed in the immunofluorescent images, a counter stain for nuclei with Hoechst (blue) was performed. Bar indicates 500 µm in phase-contrast images and 100 µm in confocal images. (For interpretation of the references to color in the text, the reader is referred to the web version of this article.)

If cardiac cell cultures are not homogeneous after hPSC differentiation, the cell culture can be purified as illustrated in Fig. 3. Using phase-contrast microscopy it becomes clear that a homogenous beating monolayer was obtained after purification. This was further confirmed by immunofluorescent staining for cardiac markers.

## Conclusion

The LM technique is a robust and easy to implement technique that can provide high quality analysis in a cost-effective manner. 3D cell cultures have been described to more closely resemble *in vivo*-like responses. Cells in a LM droplet tend to self-assemble into a spheroid structure in relatively short timeframe. In this paper, we demonstrated fumed silica powder as a LM technology to create 3D cardiospheres. After providing the cardiomyocytes with a confined environment, cardiospheres were formed within 24 h. These cardiospheres can be harvested from the LM and can be used in various downstream applications. Early outgrowth of the cardiospheres, produced with this LM technique, show that cardiac marker expression was retained in the LM. Overall, the reported method demonstrates an easy to implement technology that does not require specialized equipment or training, robustly leading to cardiospheres. The LM provides a superior alternative for 3D cell culture compared to the conventional hanging drop platform and offers great opportunities for downstream applications.

## Declaration of Competing Interest

The authors declare that they have no known competing financial interests or personal relationships that could have appeared to influence the work reported in this paper.
